# Meselect – A Rapid and Effective Method for the Separation of the Main Leaf Tissue Types

**DOI:** 10.3389/fpls.2016.01701

**Published:** 2016-11-15

**Authors:** Julia Svozil, Wilhelm Gruissem, Katja Baerenfaller

**Affiliations:** Department of Biology, Swiss Federal Institute of Technology ZurichZurich, Switzerland

**Keywords:** *Arabidopsis*, leaf, tissue, epidermis, vasculature, mesophyll, mechanical separation

## Abstract

Individual tissues of complex eukaryotic organisms have specific gene expression programs that control their functions. Therefore, tissue-specific molecular information is required to increase our understanding of tissue-specific processes. Established methods in plants to obtain specific tissues or cell types from their organ or tissue context typically require the enzymatic degradation of cell walls followed by fluorescence-activated cell sorting (FACS) using plants engineered for localized expression of green fluorescent protein. This has facilitated the acquisition of valuable data, mainly on root cell type-specific transcript and protein expression. However, FACS of different leaf cell types is difficult because of chlorophyll autofluorescence that interferes with the sorting process. Furthermore, the cell wall composition is different in each cell type. This results in long incubation times for refractory cell types, and cell sorting itself can take several hours. To overcome these limitations, we developed Meselect (mechanical separation of leaf compound tissues), a rapid and effective method for the separation of leaf epidermal, vascular and mesophyll tissues. Meselect is a novel combination of mechanical separation and rapid protoplasting, which benefits from the unique cell wall composition of the different tissue types. Meselect has several advantages over cell sorting: it does not require expensive equipment such as a cell sorter and does not depend on specific fluorescent reporter lines, the use of blenders as well as the inherent mixing of different cell types and of intact and damaged cells can be avoided, and the time between wounding of the leaf and freezing of the sample is short. The efficacy and specificity of the method to enrich the different leaf tissue types has been confirmed using *Arabidopsis* leaves, but it has also been successfully used for leaves of other plants such as tomato or cassava. The method is therefore useful for plant scientists investigating leaf development or responses to stimuli at the tissue-specific level.

## Introduction

Plant organs are composed of different tissues. Leaves are the major site of photosynthesis, and a typical dicotyledonous leaf has five major tissues. The upper tissue is formed by cells of the adaxial epidermis, followed by a single cell layer of palisade mesophyll tissue and the spongy mesophyll tissue. The spongy mesophyll is 3–4 cell layers deep and contains large air spaces around the stomata, which are embedded in the abaxial epidermis. The epidermis mainly consists of pavement and guard cells, as well as trichomes primarily on the adaxial epidermis. The vascular tissue containing the phloem and xylem forms a network between the palisade and spongy mesophyll tissues throughout the leaf lamina. The relative contributions of mesophyll, epidermal and vascular cells change during leaf development, and the final proportions in the fully expanded first leaf are about 36% each of mesophyll and vascular cells and 28% of epidermal cells (Pyke and López-Juez, 1999). An expanded leaf therefore contains a considerable number of both photosynthetically active source cells and non-photosynthetic sink cells.

Clustering of functional organ- and organelle-specific sub-proteomes separated the photosynthetically active and non-photosynthetic plant organs for plastid and peroxisomal proteins, respectively, and photosynthetic proteins were co-regulated in photosynthetic organs (Baerenfaller et al., 2008, 2011). The different photosynthetic capacities and roles of the individual leaf tissues are therefore reflected in their specific molecular profiles. Understanding tissue-specific processes requires molecular data, but these can only be generated after separation of the individual tissue types. Different techniques have already been developed depending on the cell or tissue type of interest. For example, trichomes protrude from the adaxial epidermis and therefore can be mechanically separated for small-scale proteomics experiments (Wienkoop et al., 2004; Marks et al., 2008; Van Cutsem et al., 2011). Microcapillaries have been used to collect cell sap from epidermal or S-cells for proteomics of accessible cell types (Wienkoop et al., 2004; Koroleva and Cramer, 2011). Epidermal and vascular tissues can be excised using laser capture microdissection (Nakazono et al., 2003). Any other approach to collect embedded cell or tissue types requires enzymatic digestion of cell walls to obtain protoplasts. Different enzyme concentrations and incubation times can effectively separate leaf mesophyll and guard cell protoplasts (Zhao et al., 2008; Zhu et al., 2009), while cotyledon epidermal and vascular protoplasts can be isolated using gentle sonication and manual separation (Endo et al., 2014). Fluorescence-activated cell sorting (FACS) in combination with green fluorescent protein (GFP)-marked cell type-specific enhancer trap lines has been successful for separating specific cell types from *Arabidopsis* roots for transcriptome and proteome analyses (Birnbaum et al., 2003; Brady et al., 2007; Dinneny et al., 2008; Petricka et al., 2012). FACS of leaf protoplasts is generally challenging because chlorophyll autofluorescence interferes with the sorting process (Galbraith, 2007). Nevertheless, FACS combined with GFP-expressing enhancer trap lines allowed the enrichment of guard cell protoplasts (Gardner et al., 2009), as well as leaf epidermal, vascular and guard cell protoplasts (Grønlund et al., 2012). These methods have generally low yields of cells and consequently low concentrations of RNA and proteins after extraction. They require suitable enhancer trap lines and 4–5 h from blending the plant leaves to collecting sorted protoplasts. In addition, the specificity of these methods has not been assessed and the extent of contamination with other cell types is largely unknown.

**FIGURE 1:**
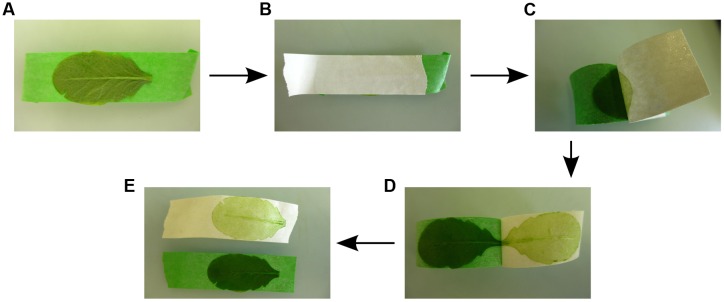
**Separation of the abaxial (lower) epidermal tissue from the remaining leaf tissues.** **(A)** The adaxial (upper) leaf side is attached to the first (green) tape strip. **(B)** The second (white) tape strip is carefully rubbed on the abaxial (lower) epidermis so that the leaf is firmly sandwiched between the two tape strips. **(C**–**E)** By gently pulling off the second (white) tape strip the abaxial epidermis is peeled off, while the remainder of the leaf tissues remains attached to the first (green) tape strip.

The Meselect (mechanical separation of leaf compound tissues) method described here effectively separates leaf epidermis, mesophyll, and vasculature tissues in about 1 h without the requirement of special plant lines or FACS equipment. Meselect first utilizes the previously reported TAPE sandwich method (Wu et al., 2009), which was developed for production of mesophyll protoplasts, to separate the abaxial epidermis from the remainder of the leaf (**Figure 1**). Mesophyll cells that remain attached to the abaxial epidermis are then removed by rapid protoplasting, which leaves the epidermal tissue intact. The tape with attached abaxial epidermis is snap-frozen in liquid nitrogen from which they can subsequently be collected using tweezers and scrapers. The remainder of the leaf attached to the other tape is incubated in protoplasting solution to release the mesophyll cells. The vascular tissue embedded in the mesophyll remains intact during this procedure and is then collected using a tweezer. Meselect is therefore a novel combination of mechanical separation and rapid protoplasting, which benefits from the differential cell wall composition of the different tissue types.

The separation efficacy and specificity of the Meselect method has been previously demonstrated using leaves from *Arabidopsis* enhancer trap lines with specific GFP expression in the epidermal, vascular and mesophyll tissues. Meselect was then successfully applied to perform tissue-specific proteome analyses after inhibition of the proteasome and affinity enrichment of ubiquitylated proteins in wild type *Arabidopsis* leaves, indicative for the high protein yield of the method (Svozil et al., 2015). Meselect has been tested using *Arabidopsis* leaves treated with proteasome inhibitor or Tween solution only. We found that the method works effectively as long as the experimental treatment does not impair leaf structures, e.g., caused by wilting or extensive lesions. We also successfully applied Meselect to separate tissues of tomato and cassava leaves and of leaves from *Arabidopsis* mutants and ecotypes, with the exception of the C24 ecotype in which the leaf structure was weakened (data not shown). Meselect is therefore likely useful for separating the main leaf tissues from most plants that produce leaves with a flattened lamina. Due to the high yield, the method is suitable for subsequent selection or enrichment steps, paving the way for studies investigating leaf development and tissue-specific processes in various plants and in response to a variety of stimuli.

## Materials and Equipment

### Reagents

•  Fully expanded rosette leaves of *Arabidopsis thaliana* ecotype Columbia. The number of required leaves depends on leaf size. Here, 10 leaves had an initial total average fresh weight of 731.7 ± 72.8 mg in six biological replicates.
•  Cellulase Onozuka RS (Yakult Honsha, cat. no. 203023);
•  Macerozyme Onozuka R-10 (Yakult Honsha, cat. no. 202030);
•  D-Mannitol (Sigma-Aldrich, cat. no. M4125);
•  MES (2-(*N*-Morpholino)ethanesulfonic acid hydrate; Sigma-Aldrich, cat. no. M 2933);
•  Potassium chloride (KCl; Carl Roth, cat. no. 6781.1);
•  Calcium chloride dehydrate (CaCl_2_ × 2 H_2_O; Sigma-Aldrich, cat. no. C3881);
•  Sodium chloride (NaCl; Carl Roth, cat. no. 3957.2);
•  Magnesium chloride hexahydrate (MgCl_2_ × 6 H_2_O; Sigma-Aldrich, cat. no. 63072);
•  BSA (Albumine, Bovine Fraction V; Carl Roth, cat. no. 8076.2);
•  Tris Base (Brunschwig, cat. no. 20092391);
•  SDS (C_12_H_15_ × NaO_4_S; Brunschwig, cat. no. 19822359);
•  Urea (Brunschwig, cat. no. GEPURE00-66);
•  Liquid nitrogen.

CAUTION: Wear safety goggles and gloves and do not touch the liquid nitrogen.

•  Protease inhibitor cocktail complete tablets (Roche, cat. no. 11.697.498.001);
•  BCA Protein Assay Kit (Thermo Fisher Scientific AG, cat. no. 23225).

### Equipment

•  Refrigerated bench-top centrifuge (5804 R, Eppendorf), set to 4°C;
•  Swing bucket centrifuge rotor for holding 15 ml falcon tubes (A-4-44, Eppendorf);
•  Bench-top microcentrifuge (5415 D, Eppendorf), set to room temperature (RT);
•  Mortar and pestle;
•  Pasteur pipettes 3 ml (Carl Roth, cat. no. EA66.1) or cut 1 ml pipette tips;
•  PES Syringe Filter, 22 μm (Brunschwig, cat. no. SFPES030022S);
•  Petri dishes 140 mm (Sarstedt AG, cat. no. 82.1184);
•  pH meter (SevenEasy pH, Mettler-Toledo);
•  Scissor;
•  Shaker (KL-2, Edmund Bühler);
•  Syringe 50 ml (Huberlab AG, cat. no. 3.7410.08);
•  Tape (Huberlab AG, cat. no. 15.5340.03) (see Note 1);
•  Tubes 15 ml (Sarstedt AG, cat. no. 62.554.502);
•  Tweezer (spade tweezer and straight tweezer);
•  Ultrospec 3000 photometer (Pharmacia Biotech).

### Solutions

•  **Protease inhibitor stock solution:** For a 25x stock solution dissolve one protease inhibitor cocktail tablet in 2 ml water, aliquot and store at -20°C. Not stable at RT.
•  **Buffer A:** 0.4 M mannitol, 10 mM CaCl_2_, 20 mM KCl, 0.1% (wt/vol) BSA, 20 mM MES, pH 5.7. Buffer A can be prepared, aliquoted, frozen and stored for 1 year at -20°C. It is stable in the refrigerator for 1 week.
•  **Enzyme solution** (always prepare fresh): 1% (wt/vol) cellulase, 0.25% (wt/vol) macerozyme, 2x protease inhibitor cocktail. Dissolve completely in buffer A, set pH to 5.7, and filter through a syringe filter. See Note 2 for how to estimate and, if necessary, reduce protease activity in the enzyme solution.
•  **Washing buffer:** 154 mM NaCl, 125 mM CaCl_2_, 5 mM KCl, 2 mM MES, pH 5.7. The washing buffer can be prepared, aliquoted, frozen, and stored for 1 year at -20°C. It is stable in the refrigerator for 1 week.
•  **SDS buffer** (always prepare fresh): 4% (vol/vol) SDS [from 10% (wt/vol) SDS stock solution], 40 mM Tris base, 2x protease inhibitor cocktail.
•  **Urea buffer** (always prepare fresh): 8 M urea (wt/vol), 20 mM Tris base, 5 mM MgCl_2_, 2x protease inhibitor cocktail.

## Stepwise Procedures

**FIGURE 2:**
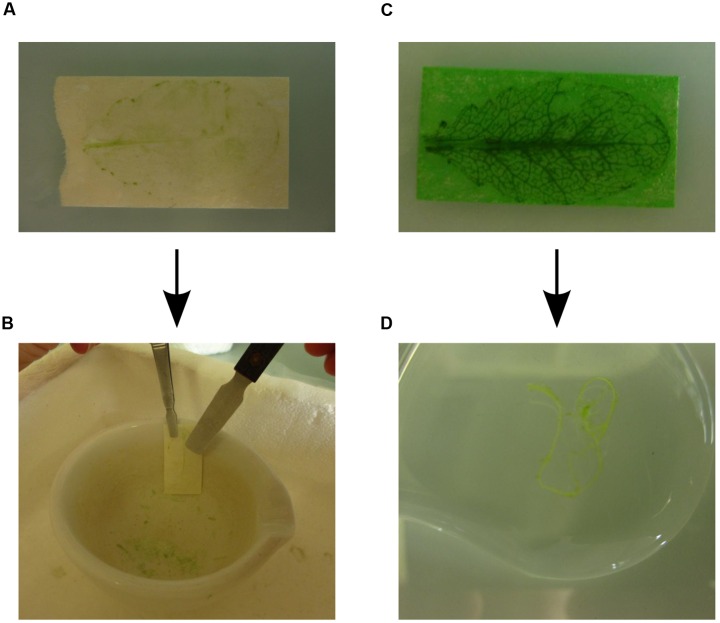
**Rapid protoplasting and collection of individual leaf tissues.** **(A)** Cleared abaxial epidermal tissue after 15 min incubation in enzyme solution. **(B)** To collect the abaxial epidermal tissue the tape strip is frozen in liquid nitrogen. The abaxial epidermis tissue is scraped off from the frozen tape strip using a scraper. **(C)** The remaining leaf vascular and adaxial epidermal tissues after 45–60 min incubation in enzyme solution, which released the mesophyll cells into the enzyme solution. **(D)** The vasculature is collected with a tweezer and placed in washing buffer.

(1) Prepare buffer A, enzyme solution and washing buffer before starting the protocol (for 10 leaves prepare 10 ml buffer A, at least 30 ml enzyme solution and 100–200 ml washing buffer). The washing buffer needs to be ice-cold. Distribute the enzyme solution into two Petri dishes. Prepare an extra Petri dish with 10 ml buffer A and five Petri dishes labeled W1–W5, which are used for the washing steps.
Cut and set up the tape strips as indicated in **Figure 1**. Using two differently colored tapes helps to distinguish between abaxial (lower) epidermis fixed to one tape strip and the remainder of the leaf fixed to the other tape strip. Bend one end of the first tape strip (here, green tape strip) and fix it on the bench, such that the adhesive side of the tape strip is facing upward.
**CRITICAL:** We recommend not wearing gloves for steps 2 and 3 because they stick to the tape and it is difficult to remove one of the tapes wearing gloves.
(3) Harvest the leaf and attach the upper leaf side with the adaxial epidermis to the green tape strip that is fixed on the bench (**Figure 1A**). Place the second tape strip (here, white tape strip) on the lower leaf side with the abaxial epidermis and rub it down carefully so that the leaf is firmly sandwiched within the two tape strips (**Figure 1B**). Remove the abaxial (lower) epidermis by gently pulling off the white tape. The abaxial epidermis will remain attached to the tape without the vasculature and with only few spongy mesophyll cells (**Figures 1C,D**) (see Note 3 of how to fix leaves that are larger than the tape strips, Note 4 of how to handle incomplete separation of the tissues, and Note 5 of how to proceed if the vascular tissue remains attached to the abaxial epidermis). Cut the edges of both tape strips to the size of the leaf. Place the green tape strip with the remainder of the leaf tissues in the Petri dish with enzyme solution and store the white tape strip with the abaxial epidermis in buffer A. Repeat the procedure for up to 10 leaves, which takes 5–30 min depending on practical experience.
**CRITICAL:** The separation of the abaxial epidermis from the remaining leaf tissues is the most critical step of the whole procedure. Therefore, we recommend practice tests with non-critical leaf material first.
(4) Transfer all the white tape strips with the abaxial epidermis at once from buffer A to enzyme solution using a tweezer. This synchronization is necessary to ensure that the abaxial epidermis is cleared from contaminating mesophyll cells but no epidermal cells are released into the enzyme solution. Incubate at RT on the shaker at 50 rpm for 15 min.
(5) Visual inspection of the abaxial epidermis after 15 min of incubation should reveal that the tissue layer attached to the tape strip has lost much of the green color because the abaxial epidermis is now largely cleared of spongy mesophyll cells (**Figure 2A**). Pour 15 ml washing buffer each into Petri dishes W1 and W2 and wash the white tape strips with attached abaxial epidermis twice by transferring them first to W1 and then to W2. Let the tape strips drip off quickly and remove excess washing buffer with paper cloth without touching the epidermis. Directly freeze the tape with the attached epidermis in liquid nitrogen and store at -80°C. Discard washing and enzyme solutions.

(6) The green tape strips with the remaining leaf tissues are incubated at RT in enzyme solution on the shaker at 30–50 rpm for 45–60 min. Cell wall digestion can be followed visually because the release of mesophyll protoplasts into solution reduces the green color of the epidermis tissue attached to the tape strip while the green color of the solution becomes more intense. Monitoring of cell wall digestion must begin 35 min after the start of incubation because the activities of cellulases and macerozymes vary and protoplasting time depends on leaf type and age. For instance, releasing protoplasts from younger leaves takes more time than from older leaves. After 45 min most mesophyll protoplasts will be released into the enzyme solution and only the upper epidermis and the vasculature remain attached to the tape strips (**Figure 2C**).
(7) After release of the mesophyll protoplasts into enzyme solution, place the tape strips with the remaining adaxial leaf epidermis and vascular tissues in Petri dish W3 containing cold washing buffer. The vasculature tissue can now be removed gently using tweezers and is placed in Petri dish W4 with cold washing buffer (**Figure 2D**). After washing the vasculature once again in Petri dish W5 containing cold washing buffer, freeze it directly in liquid nitrogen and store at -80°C.
(8) Collect the enzyme solution containing the mesophyll protoplasts in a 15 ml falcon tube. In order to minimize protoplast breakage, use a Pasteur pipette with a wide opening or cut a regular 1 ml tip and pipette slowly.
(9) Pellet the mesophyll protoplasts by centrifugation at 100 *g* for 4 min at 4°C, remove the supernatant and wash the pellet with 10 ml cold washing buffer. Repeat once. Remove the supernatant and freeze the mesophyll protoplasts directly in liquid nitrogen and store at -80°C.
**PAUSE POINT:** Frozen mesophyll protoplasts, vascular tissue and epidermis attached to tape can be stored at -80°C until further processing.
(10) Remove abaxial epidermis from the tape strips. Place a mortar and pestle in liquid nitrogen and wait until boiling of nitrogen ceases. Grip one frozen tape strip with a spade tweezer, hold it against the wall of the mortar and use a cold tweezer and/or scraper to remove the epidermis from the tape (**Figure 2B**). After removal of the epidermis from all tape strips, directly grind the tissue in the mortar. Collect the tissue powder in an Eppendorf tube.
**CRITICAL:** The tape glue loses its adhesive property below -160°C, but static forces keep the epidermis attached to the tape. For this reason the scraper is needed to remove the epidermis.
(11) Grind the mesophyll cells and vascular tissue with mortar and pestle in liquid nitrogen. Collect the tissue powders in Eppendorf tubes.
**PAUSE POINT:** The ground epidermal, vascular and mesophyll tissue powders can be stored at -80°C until further processing.
(12) Prepare the protein extraction buffer (either SDS or urea buffer) immediately before starting the extractions.
(13) After separating and grinding the tissues of 10 leaves, add 200 μl protein extraction buffer to the epidermal tissue powder, 500 μl to the mesophyll tissue powder and 100 μl to the vascular tissue powder (see Note 6 for extraction of RNA).
(14) Vortex and incubate the Eppendorf tubes for 20 min at RT before centrifugation in the microcentrifuge at 16,400 *g* (or maximal speed) for 10 min at RT.
(15) Collect the supernatants and transfer to new Eppendorf tubes. Discard the pellets. Protein concentration in the individual tissue extracts can be determined using the BCA Protein Assay Kit.
**CRITICAL:** The protein extracts must be immediately processed further. They cannot be frozen or stored at RT for an extended time because proteins will precipitate especially in epidermal tissue extracts.

**TIMING**
Steps 1 and 2: 30 min;
Step 3: 5–30 min;
Steps 4 to 9: 60–80 min;
Step 10: 20 min;
Step 11: 10 min;
Steps 12 to 14: 40 min.

## Anticipated Results

**FIGURE 3:**
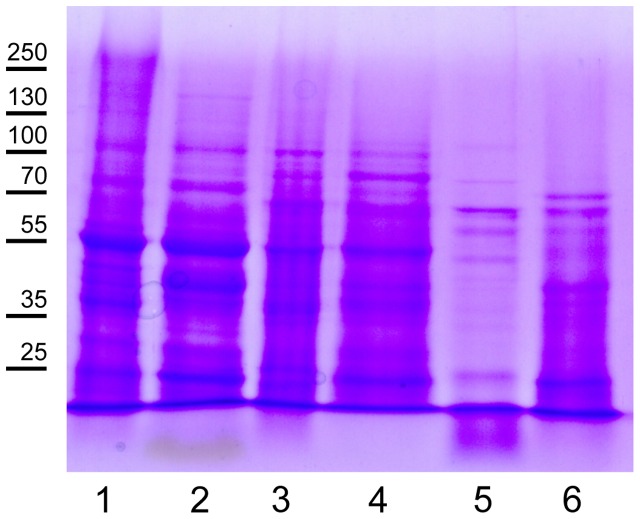
**Representative protein pattern of leaf tissue extracts.** Lanes 1, 3, and 5: Urea and lanes 2, 4, and 6: SDS protein extracts of separated mesophyll (lanes 1, 2), vascular (lanes 3, 4) and epidermal tissues (lanes 5, 6). Proteins were separated in a 10% SDS PAGE gel and stained with Coomassie Brilliant Blue. Each lane was loaded with 20 μg of protein. Molecular weight markers are indicated on the left. The prominent band in lanes 1 and 2 at an apparent molecular mass of 55 kDa is RBCL.

Protein concentrations in the SDS and urea tissue-specific extracts were determined with the BCA Protein Assay Kit, which is compatible with high SDS concentrations. The typical protein yields for 10 fully expanded *Arabidopsis* leaves are shown in **Table 1**. Compared to the protein/fresh weight (FW) ratio of around 0.01 for fully expanded leaves, the tissue-specific extracts have lower ratios with lowest values for vascular tissue. Electrophoretic separation of proteins in the tissue-specific extracts should result in patterns similar to those shown in **Figure 3**. In the mesophyll tissue protein extract the large subunit of RUBISCO (RBCL) is the most prominent band at 53 kDa. If this is not the case, the protease activity in the cellulose and macerozyme enzyme solution might be too high and needs to be reduced (see Note 2). In contrast to the mesophyll tissue protein extract in which few abundant proteins form prominent bands, the protein bands in the vascular and especially the epidermal tissue extracts are more evenly distributed.

**Table 1:** Protein yield of the Meselect method.

Tissue	Extraction buffer	Total protein [mg]	Protein/FW [mg/g]
Mesophyll	SDS	5.07 ± 0.32	7.09E-03 ± 8.83E-04
	Urea	3.32 ± 0.42	4.51E-03 ± 7.65E-04
Vasculature	SDS	0.115 ± 0.018	1.62E-04 ± 4.53E-05
	Urea	0.070 ± 0.028	9.45E-05 ± 3.39E-05
Epidermis	SDS	0.70 ± 0.25	9.70E-04 ± 3.49E-04
	Urea	0.520 ± 0.067	7.02E-04 ± 4.90E-05


*Protein yield of mesophyll, vascular and epidermal tissues after tissue separation of 10 fully expanded *Arabidopsis* rosette leaves, protein extraction with SDS or urea extraction buffer, and protein quantification using the BCA Protein Assay Kit. Values are the means of six biological replicates with standard deviations.*

To assess the efficacy of separating the different tissue types, the respective protein extracts can be analyzed by Western Blot or mass spectrometry-based proteomics to determine the enrichment of proteins in the extract of a specific tissue compared to a complete leaf extract and correspondingly their depletion in extracts from other tissues. **Supplementary Table S1** contains tissue-specific proteins extracted from lists of proteins only identified in one tissue type (Svozil et al., 2015). The lists were additionally filtered for proteins that were identified with at least 10 tandem mass spectra in the respective tissue type and that were not identified after ubiquitin affinity enrichment to exclude proteins that might only accumulate after inhibition of the proteasome. These tissue-specific proteins are therefore useful markers that should be readily identified in protein-based assays. Alternatively, RNA can be extracted from the tissue powders in step 13 of the Stepwise Procedures. The enrichment of transcripts for tissue-specific proteins in a specific tissue type can then be tested using quantitative RT-PCR (qRT-PCR). However, when interpreting the qRT-PCR data it needs to be considered that mRNA and protein levels are not necessarily correlated (Baerenfaller et al., 2012).

## Notes

**FIGURE 4:**
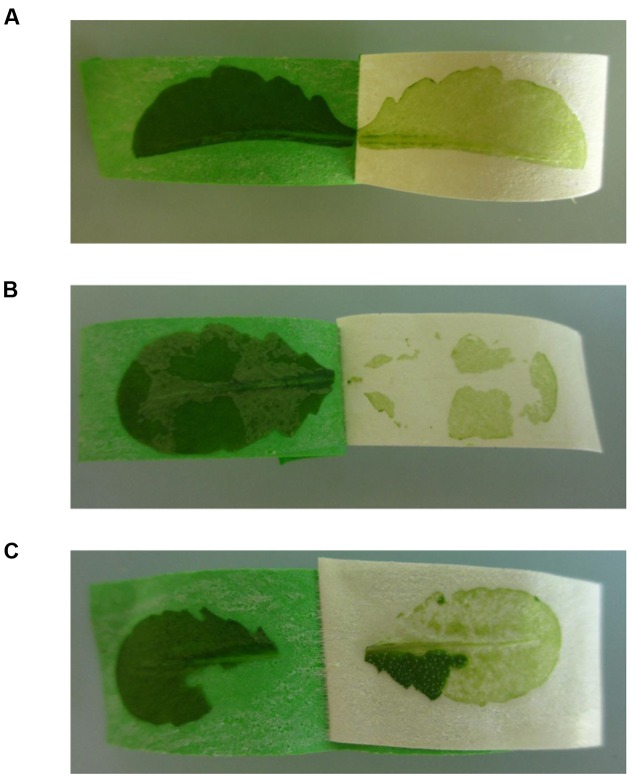
**Possible problems that can be encountered during the Meselect tape sandwich method (see Notes 3 to 5).** **(A)** Leaves that are wider than the tape strips are cut lengthwise into two halves. **(B)** Patches of abaxial epidermal tissue that remain attached to the remaining leaf tissues. **(C)** Patches of leaf tissues that remain attached to the abaxial epidermis.

(1) **Tape properties:** Not every tape brand is suitable for performing the tape sandwich tissue separation. Some tape brands do not adhere strongly enough to the leaf. It is therefore recommended to test different tape brands in case the tape brand we have listed is not available.
(2) **Protease activity in cellulose and macerozyme enzyme solution:** The cellulose and macerozyme enzymes are supplied as crude enzyme powders. Depending on the batch they contain varying amounts of proteases, which in extreme cases can result in almost complete protein degradation, especially during the preparation of mesophyll protoplasts. We therefore recommend to test each enzyme batch before use and, if necessary, to reduce protease activity. To estimate protease activity in the enzyme solution, prepare protein extracts from mesophyll protoplasts and total leaf tissue, and separate the proteins using SDS PAGE. After staining of the gel with Coomassie Brilliant Blue, the apparent RBCL molecular mass of 53 kDa is compared between the different extracts. In the total leaf extract RBCL is a prominent band at around 55 kDa. If protease activity in the enzyme solution is very high, the intensity of the RBCL band is decreased or has disappeared. If protease activity is low RBCL may migrate with a smaller apparent molecular mass and other prominent protein bands become visible in the lower mass range of the gel. If protease activity in the enzyme solution is high, it needs to be reduced as described in (Fairley-Grenot and Assmann, 1992; Miedema and Assmann, 1996) by lowering the pH of the enzyme solution to 3.5 for 10 min using HCl, subsequently increasing the pH again to 5.7 using KOH, and adding protease inhibitor cocktail to a final concentration of 2x. Heating the enzyme solution to 55°C for 10 min in our experience has no effect on protease activity. Since acidification of the enzyme solution also reduces the activity of the cell wall-degrading enzymes, their concentrations can be increased up to 4% (wt/vol) cellulase and 1% (wt/vol) macerozyme for effective protoplasting.
(3) **Handling of large leaves:** In case the leaves are wider than the tape strips, cut the leaves along the middle vein (**Figure 4A**). You can use both leaf halves, but it is more difficult to collect the vascular tissue from the leaf half without the middle vein.

(4) **Incomplete separation of the abaxial epidermis from the remaining leaf tissues:** Occasionally patches of the abaxial epidermis remain attached to the spongy mesophyll cells (**Figure 4B**) or patches of the leaf remain stuck on the abaxial epidermis when the tape strips are pulled apart (**Figure 4C**). To remove these tissue patches you can use a new tape strip, or the same tape strip in case you have not yet pulled the tape strips completely apart. The tissues are especially difficult to separate at the edges of the leaf. Here, a new tape strip is needed to remove residual patches.
(5) **Vascular tissue remains attached on the abaxial epidermis:** When using leaves that are particularly thin it may happen that the vascular tissue remains attached to the abaxial epidermis instead of being embedded in the remaining leaf tissues. In this case proceed to steps 4 and 5 to remove contaminating mesophyll cells, but collect the vascular tissue as described in step 7 before freezing the tape with the attached abaxial epidermis.
(6) **Extraction of RNA:** The ground tissue powders can also be used to extract RNA in addition to proteins, or instead of proteins. RNA can be extracted from an aliquot or the total tissue powders using standard RNA extraction procedures either with the RNeasy Plus Mini kit (Qiagen), TRIzol (Invitrogen) or TRI (Sigma-Aldrich).

## Author Contributions

JS and KB designed research; JS performed research; JS, WG, and KB wrote the paper and approved the final version to be published.

## Conflict of Interest Statement

The authors declare that the research was conducted in the absence of any commercial or financial relationships that could be construed as a potential conflict of interest.
